# Beneficial effects of exercise on chemotherapy-induced peripheral neuropathy and sleep disturbance: A review of literature and proposed mechanisms

**DOI:** 10.1016/j.gore.2022.100927

**Published:** 2022-01-07

**Authors:** L. Brett Whalen, W. Zachary Wright, Priyanka Kundur, Siddhartha Angadi, Susan C. Modesitt

**Affiliations:** a University of Virginia School of Medicine, Charlottesville, VA, United States; b Department of Kinesiology, School of Education and Human Development, University of Virginia, Charlottesville, VA, United States; c Division of Gynecologic Oncology, Department of Obstetrics and Gynecology, School of Medicine, University of Virginia, Charlottesville, VA, United States

**Keywords:** Exercise, Chemotherapy, Peripheral neuropathy, CIPN, Sleep, Cancer

## Abstract

The goal of this narrative review is to evaluate the literature regarding exercise training as a therapy to prevent or mitigate deleterious side effects of chemotherapy, specifically peripheral neuropathy and sleep disturbances and to make concrete recommendations for implementation for the practicing oncologist. A literature search was conducted for studies that included an exercise intervention to be implemented for patients undergoing or previously treated with chemotherapy along with an analysis of its effect on either chemotherapy-induced peripheral neuropathy (CIPN) or chemotherapy-induced sleep disturbances. Studies were subsequently analyzed and summarized in order to determine the overall promise of exercise as a therapy in this setting. Five studies met inclusion criteria to be assessed with regard to the effect of exercise on CIPN and eight were included for sleep disturbances. Exercise was found to be a significantly beneficial therapy in preventing, mitigating, or improving the symptoms of CIPN and sleep disturbances in cancer patients in the majority of studies evaluated. Exercise is an effective intervention and should be specifically prescribed concurrently with chemotherapy to maximize potential of avoiding these debilitating side effects, which significantly and negatively impact quality of life in cancer survivors.

## Research highlights

• Exercise counters chemotherapy-induced peripheral neuropathy and sleep disturbances.
• Exercise reduces inflammation, promotes neuroplasticity, modulates pain signaling.
• Exercise improves sleep disturbances by entraining circadian rhythmicity.
• Oncologists should learn how to prescribe exercise regimens for their patients.

## 1 Introduction

Over the past few decades, improvements in cancer treatments have prolonged survival, but patients often have to live with long-term treatment related side effects, two of the most prevalent are peripheral neuropathy (Duregon et al., 2018) and sleep disturbances (Palesh et al., 2012). Chemotherapy-induced peripheral neuropathy (CIPN) affects 30–60% of patients treated with neurotoxic agents like paclitaxel (Duregon et al., 2018), and persistent symptoms are present in nearly 40% of cancer survivors (McCrary et al., 2019). The hands and feet are most often affected, resulting in numbness, tingling, neuropathic pain, cramping, and difficulties with balance. Orthostatic hypotension, which arises from involvement of autonomic neurons, is another common manifestation (Kleckner et al., 2018). Currently, there are no FDA-approved therapies for CIPN (Kleckner et al., 2018) nor satisfactory evidence to recommend alternative therapies such as acupuncture, physical therapy, or capsaicin ointment (Fernandes and Kumar, 2016).

Sleep disturbances, which affect between 30 and 87% of cancer patients, are also reported during chemotherapy and can similarly persist for years following treatment termination (Palesh et al., 2012). Commonly reported problems include frequent night awakenings (49–56% of patients), inability to fall asleep (50–73%) and early awakenings (49–65%) (Palesh et al., 2012). Importantly, poor sleep quality has been found to correlate with decreased quality of life (QOL) and worsening depression indices in patients with ovarian, fallopian tube, or primary peritoneal cancers (Palesh et al., 2012), and has also been identified as a negative prognostic indicator for overall survival in women receiving chemotherapy for ovarian cancer (Sandadi et al., 2011).

Peripheral neuropathy and sleep disturbances can be long lasting, negatively affect QOL, and lack easy remedies that physicians can offer to their patients who are suffering. Therefore, it is worthwhile to explore all potential avenues to prevent or relieve these side effects, including medication and lifestyle interventions. While most drug therapies have failed to demonstrate efficacy in this regard, exercise therapy stands out as a potential protective or mitigating factor. Physical activity has been proven to decrease both all-cause mortality and all-cancer mortality risk, as well as the risk of over 25 chronic medical conditions including cardiovascular disease, type 2 diabetes, breast cancer, and colon cancer (Warburton and Bredin, 2017) but has been underutilized by treating physicians, likely due to difficulty in knowing the exact exercise prescription/intervention to prescribe. Similarly, high cardiorespiratory fitness (VO2peak) is associated with lower all-cause and cancer mortality risk and importantly, this effect is independent of overweight/obesity status (Schmid and Leitzmann, 2015).

The benefits of exercise have also been described in the domains of peripheral nerve health and sleep. For example, in animal models, exercise reduces hyperalgesia and allodynia, while observational studies in humans suggest that people who engage in more physical activity at baseline carry lower risk of later developing peripheral neuropathy (Leitzelar and Koltyn, 2021). Exercise has also been shown to improve sleep problems in the general population (Palesh et al., 2012). In a cross-sectional study of 339 midlife women in which sleep was examined with questionnaires, diaries, and in-home polysomnography, Kline et al. found that women who had a historical pattern of greater sports/exercise activity (but not lifestyle- or household-related activity) had better Pittsburgh Sleep Quality index scores and higher sleep efficiency as assessed by polysomnography (Kline et al., 20132013). Interestingly, a correlation between CIPN severity and sleep quality has been demonstrated, such that higher CIPN symptom scores were found to be associated with poorer sleep quality (Hong et al., 2014). When taken together, the evidence supporting exercise as a medical therapy is overwhelmingly positive and wide-reaching, yet exercise as a treatment modality remains under-prescribed and under-appreciated.

**Fig. 1:**
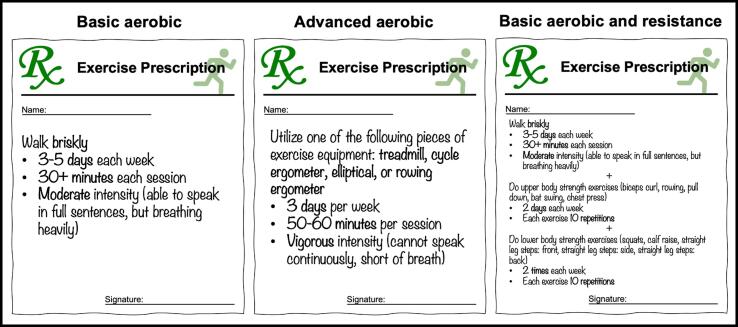
**Specific exercise programs for clinicians to prescribe for patients.***Prescription 1* (Wang et al., 2011 Mar) (Tang et al., 2010) (Wenzel et al., 20132013) (“Basic Aerobic”) may be appropriate for patients with limited exercise tolerance, experience, or motivation to start a more complex regimen. *Prescription 2* (Courneya et al., 2012) (Courneya et al., 2014) (“Advanced Aerobic”) may be appropriate for patients with more robust exercise capacity. *Prescription 3* (Cheville et al., 2013) (“Basic Aerobic Plus Resistance”) may be appropriate for highly motivated patients who have limited exercise experience or tolerance. All three regimens can be completed at home with little requirement for upfront coaching or access to equipment. Detailed descriptions of the exercises in Prescription 3 can be found in Supplementary Materials 1.

The “Exercise is Medicine” movement has swept through the clinical realm, raising new possibilities for the application of exercise in the management of historically difficult-to-treat chemotherapy side effects. This may be especially important for patients with ovarian cancer, most of whom reduce their physical activity during treatment such that only a small minority attain the 150 min of moderate-intensity exercise per week recommended by the American College of Sports Medicine (ACSM) (Sandadi et al., 2011). One barrier to implementation may stem from a lack of practical knowledge regarding how to specifically prescribe concrete and actionable exercise regimens for every patient (see Fig. 1 for potential prescriptions). The goals of this review were to elucidate and summarize the role of exercise interventions in the treatment or prevention of CIPN and chemotherapy-related sleep disturbances, to describe proposed mechanisms, and to suggest next steps in research and implementation that will allow the field of Gynecologic Oncology to leverage this natural remedy.

## 2 Exercise as a therapy for CIPN

Available research on the utilization of exercise to both prevent and treat CIPN is heterogeneous with respect to populations studied, as well as exercise modality, duration, and timing; however, the body of evidence is collectively encouraging (Table 1).

**Table 1:** Summary of included studies investigating the effects of an exercise intervention on CIPN. Abbreviations: CG, Control Group; IG, Intervention Group.

**Study**	**Participants**	**Study Design**	**Exercise Protocol**	**CIPN measures**	**Outcomes**
Henke et al., 2014	Lung cancer patients, ongoing chemotherapy (N = 46) IG: N = 25, CG: N = 21	Randomized controlled trial (RCT), outcomes measured at baseline and after 3 cycles of chemotherapy	Type: supervised hallway/stair walking and resistance training Frequency: 5 days/week & every other day (total duration of treatment) Intensity: 55–70% of heart rate reserve (HRR) for walking and 50% of maximum repetitions (RM) for strength Time: 6-minute hallway walk, 2-minute stair walk, strength sessions based on repetitions	Objective: none Subjective: European Organization for Research and Treatment of Cancer Quality of Life Questionnaire Core-30 (EORTQLQ C-30) with peripheral neuropathy sub-scale	Significant difference between groups in subscale of peripheral neuropathy (p = 0.05)
Kleckner et al. (2018)	Non-leukemia cancer without metastases, chemo-naïve, starting chemo (N = 355) IG: N = 170 CG: N = 185	RCT, outcomes measured at baseline and after 6 weeks	Type: ACSM Exercise for Cancer Patients (EXCAP): Home-based walking + resistance bands Frequency: daily Intensity: low-to-moderate Walking: 60–85% of HRR Bands: Rated perceived exertion (RPE) of 3–5 (1–10 scale) Time: individually tailored, progressive prescription based on baseline ability	Objective: none Subjective: symptoms of numbness and tingling, hot/coldness in hands/feet (0–10 scales)	Exercise associated with smaller pre- to post-intervention increases in CIPN severity. Numbness/tingling increased by 0.58 and hot/coldness increased by 0.77 in the CG while numbness/tingling increased by 0.38 and hot/coldness increased by 0.38 in the IG (p = 0.061 and p = 0.045)
McCrary et al. (2019)	Cancer survivors (≥ 3 months post-treatment with neurotoxic chemotherapies) with established CIPN symptoms. N = 29	Single-group pre-post design, outcomes measured at 3 timepoints: before 8-week control period (T0), after control period but before exercise intervention (T1), and after 8-week exercise intervention (T3)	Type: Individualized prescription of aerobic, resistance, and balance training sessions (half supervised, half done at home) Frequency: 3 sessions/week (total of 8 weeks) Intensity: all exercises performed at RPE of 13–15 (6–20 scale) Time: 1 h per session	Objective: Total Neuropathy Score- clinical version (TNSc; assessment of muscle weakness, numbness/tingling, pinprick, vibration, tendon reflex, and strength, scored 0–24), nerve conduction studies Subjective: EORTC CIPN-20 (patient-reported questionnaire of CIPN symptoms, scored 0–100)	Exercise improved clinically-assessed (TNSc baseline: 6.7, pre-exercise: 7.0, post-exercise: 5.3, *p* < 0.01) and patient-reported (CIPN-20 baseline: 26.6, pre-exercise: 25.4, post-exercise: 18.2, *p* < 0.01) symptoms of CIPN after no such improvement was found during the control period. Exercise did not significantly change sensory or motor nerve amplitudes, refractoriness, or excitability
Streckmann et al. (2014)	Lymphoma patients undergoing chemotherapy (N = 61) IG: N = 30 CG: N = 31	RCT, outcomes measured at 4 timepoints: prior to chemotherapy (T0), after 12 weeks (T1), after 24 weeks (T2), and after 36 weeks (T3)	Type: supervised aerobic (treadmill or bike-dynamometer), sensorimotor (4 postural stabilization tasks), and resistance (4 exercises) Frequency: 2 sessions/week (total of 36 weeks) Intensity: Aerobic: 70–80% HR_max_ Sensorimotor: progressively increasing task difficulty Strength: exercises carried out at maximum force Time: 1-hour sessions, each consisting of: Aerobic: 10–30 min Sensorimotor: three 20-second sets for each exercise	Objective: PNP-related deep sensitivity evaluated by tuning fork (0–8 scale), balance control on static and dynamic surfaces Subjective: EORTC QLQ-30 (quality of life questionnaire)	PNP-related deep sensitivity declined in 7/8 (87.5%) of intervention group, compared to 0/12 (0%) in control group (*p* < 0.001) IG showed greater improvements in static (*p* = 0.03) and dynamic (*p* = 0.007) balance control than CG, which showed steady decline IG reported significantly better QOL at 12 weeks compared CG (p = 0.03), though no difference between groups was seen at 36 weeks
Zimmer et al. (2018)	Stage IV colorectal cancer patients, life expectancy ≥ 6 months, undergoing palliative chemotherapy (N = 30) IG: N = 17 CG: N = 13	RCT, outcomes measured at 3 timepoints: baseline (T0), after 8-week intervention (T1), and 4 weeks post-intervention (T2)	Type: Supervised aerobic (walking, bicycle ergometer, or cross-trainer), resistance (circuit training of bench press, lat pulldown, leg press, seated row, and abdominal exercise), and balance Frequency: 2 sessions/week (total of 8 weeks) Intensity: Aerobic: 60–70% HR_max_ Resistance: 60–80% of hypothetical 1RM Time: 60-minute sessions	Objective: balance, hypothetical 1RM, endurance capacity (6MWT) Subjective: Trial Outcome Index (TOI) of the Functional Assessment of Cancer Therapy/Gynecologic Oncology Group Neurotoxicity (FAACT/GOG-NTX) questionnaire (4-point change signifies clinical significance)	Overall TOI remained stable for IG from T0 to T1 and from T0 to T2, but worsened for CG from T0 to T1 (-7.1 points, *p* = 0.028) and from T0 to T2 (-8.1 points, *p* = 0.037). Across the 8-week intervention, neuropathic symptoms significantly improved in IG (+2.12 points on NTX subscale, *p* = 0.023), while worsening in CG (-5.11 points, *p* = 0.45). Change in severity of neuropathic symptoms was significantly different between IG and CG from T0 to T1 (*p* = 0.002) and from T0 to T2 (*p* = 0.015).

With regard to exercise as a preventative intervention during chemotherapy, the largest trial to date by Kleckner et al. investigated the role of exercise in the development and progression of CIPN in 355 chemotherapy-naive cancer patients (79% of whom had breast cancer) receiving taxane-, platinum-, or vinca alkaloid-based chemotherapy (Kleckner et al., 2018). Patients were randomized to receive either chemotherapy alone or chemotherapy plus a 6-week home-based exercise program that included progressive walking and resistance training regimens. The exercise intervention was associated with smaller increases in symptom severity, however the magnitude of effect was of questionable clinical significance. On 10-point scales, numbness/tingling and hot/coldness in hands/feet increased by 0.58 and 0.77, respectively, in the control group, compared to 0.38 and 0.38, respectively, in the exercise group (*p* = 0.061 and *p* = 0.045). Post-intervention prevalence rates of numbness/tingling and hot/coldness were also lower in the exercise group than in the control group (36.5% vs. 49.2% and 33.5% vs. 45.4%, respectively), however group-specific baseline values were not reported, making it challenging to deduce the effect of the exercise intervention on CIPN prevalence rates. These findings support a modest role for exercise in attenuating the progression of CIPN symptoms and a possible role in preventing their onset.

Exercise can also help stabilize symptoms related to CIPN when initiated during subsequent chemotherapy lines. In a study of 30 patients undergoing palliative chemotherapy (median of 23.5 chemotherapy cycles of various regimens prior to intervention), Zimmer et al. randomized participants to either an 8-week supervised exercise program involving endurance, resistance, and balance training, or to a control group given written standard recommendations on physical fitness. They found that symptoms of peripheral neuropathy remained stable throughout the course of the study in the intervention group but significantly worsened in the control group. Intriguingly, they also found that changes in neuropathic symptoms correlated with changes in advanced static balance. While the authors of this study suggested that the balance component of their intervention may have played an important role in counteracting CIPN (Zimmer et al., 2018), the converse scenario is also plausible, in which stabilization of nerve health resulted in preservation of proprioceptive inputs and therefore balance. In another study involving a similar population, 20 patients with advanced stage lung cancer undergoing palliative platinum-based chemotherapy were randomized to either conventional physical therapy (control group) or an endurance, strength, and ventilatory training intervention. Following the intervention, patients in the exercise group were found to have significantly less severe peripheral neuropathy symptoms compared to the control group, along with better physical functioning, ability to perform activities of daily living, and quality of life (Henke et al., 2014). However, patients in the exercise group had five-fold lower peripheral neuropathy symptoms at baseline as well, making it difficult to discern the effect of exercise. In another trial of 61 patients with lymphoma undergoing unspecified chemotherapy, participants were randomized to either standard clinical care with physical therapy alone or in combination with a 36-week supervised exercise program involving sensorimotor, strength, and endurance training. Throughout the course of the study, total incidence of peripheral neuropathy among patients receiving neurotoxic chemotherapy was 40% (8/20) in the exercise group and 71% (12/17) in the control group. Subsequent symptomatic improvement, as determined by tuning fork assessment, was significantly better and only demonstrated in the exercise group (87.5% vs. 0%, p < 0.001) (Streckmann et al., 2014).

**Fig. 2:**
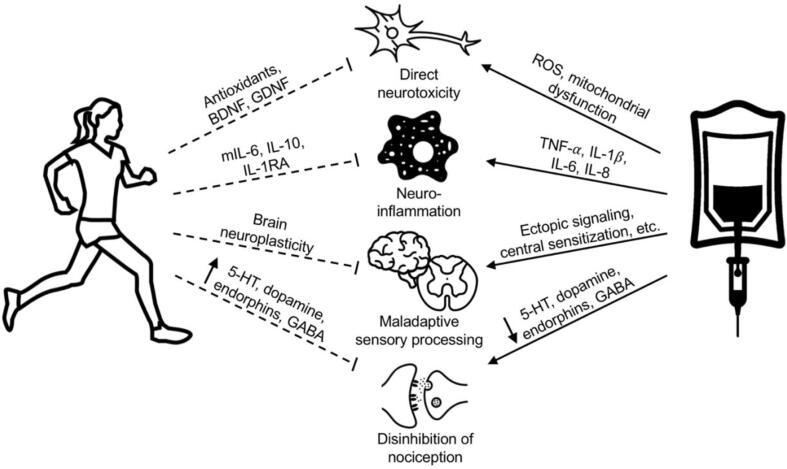
**Overview of proposed mechanisms underlying beneficial effects of exercise on CIPN. **Several biological mechanisms have been proposed to explain the relationships between exercise, CIPN, and sleep. In general, exercise counteracts the deleterious effects of chemotherapy on the nervous system by reducing inflammation, promoting brain neuroplasticity, and enhancing pain modulation pathways. Abbreviations: 5-HT, 5-hydroxytryptamine (serotonin); BDNF, brain-derived neurotrophic factor; GABA, gamma-aminobutyric acid; GDNF, glial-derived neurotrophic factor; IL-1RA, interleukin 1 receptor antagonist; IL-1B, interleukin 1Beta; IL-6, interleukin 6; IL-8, interleukin 8; IL-10, interleukin 10, mIL-6, myokine ROS, reactive oxygen species; TNF-A, tumor necrosis factor-Alpha.

For patients already suffering from the effects of CIPN, other studies provide further evidence for the ability of exercise to improve established symptoms. McCrary et al. (2019) assessed the response of CIPN symptoms to an exercise intervention in a group of 29 patients who were at least 3 months removed from their last treatment with neurotoxic chemotherapy. In this study, which employed a single-group, quasi-experimental, pre-post design, patients were evaluated for CIPN symptom severity at three time points: baseline, after an 8-week control period prior to initiating exercise, and one last time after completing an 8-week exercise program. The exercise intervention included supervised and at-home sessions of endurance, resistance, and balance training. No changes were observed during the control period, whereas following completion of the exercise program, significant improvements were seen in both patient-reported and clinically assessed CIPN symptoms, as well as dynamic and standing balance tests. However, sensory and motor excitability, tested through nerve conduction studies, were not significantly affected by the exercise intervention (McCrary et al., 2019). This study shows subjective and clinically-evident improvement in CIPN symptoms after exercise. However, the results were not corroborated by objective measures, raising the possibility that mechanisms underlying exercise-mediated symptomatic improvement in CIPN are not those that would be detected on nerve conduction studies (McCrary et al., 2019), for example central nervous system adaptations (Fig. 2).

While the extent of research investigating the effects of exercise therapies on CIPN is modest and heterogeneous, the majority of available evidence demonstrates a positive effect in either preventing, mitigating, or treating chemotherapy-induced peripheral neuropathy. Professional societies, such as the American Society of Clinical Oncology (ASCO) and European Society for Medical Oncology (ESMO), have yet to make strong recommendations on the clinical use of exercise in this context. In its most recently updated guideline, ASCO acknowledges that “preliminary supportive evidence” exists in favor of exercise to prevent and treat CIPN, but concludes that “no recommendation can be made” due to the lack of robust evidence (Loprinzi et al., 2020). ESMO, on the other hand, has taken the slightly stronger stance that exercise “can be offered” due to early reports of its protective effect (Jordan et al., 2020). Given the many well documented health benefits of exercise and reassuring safety results from the trials included in this review, we believe that the benefits of prescribing exercise to this population largely outweigh the risks and we therefore recommend this clinical practice. The key questions for clinicians are first, what to recommend for a specific exercise regimen, and second, how to proactively prescribe it to their patients to maximize uptake, compliance, and results (Fig. 1).

## 3 Exercise as a therapy for sleep disturbances

A variety of exercise programs, including aerobic and resistance modalities, have been investigated as therapies for poor sleep quality in cancer patients undergoing chemotherapy (Table 2). Wang et al. found that women with newly diagnosed breast cancer randomized to a 6-week walking program while undergoing chemotherapy reported significantly less sleep disturbance and higher quality of life over the course of their treatment compared to controls (Wang et al., 2011 Mar). Utilizing a similar intervention in a heterogeneous group of cancer patients, 14% of whom were actively receiving chemotherapy, Tang et al. found that supplementing standard care with an 8-week home-based walking program significantly improved self-reported sleep quality scores. Furthermore, the benefits in sleep were associated with reduced bodily pain and higher mental health ratings on a QOL questionnaire (Tang et al., 2010). In this study, only a minority of patients were undergoing chemotherapy, making it difficult to discern an effect of exercise on sleep disturbances that were specifically induced by chemotherapy. To this end, Courneya et al. performed a two-armed trial involving a 12-week aerobic exercise program using cycle ergometers in 122 lymphoma patients. Patients were stratified by type of lymphoma and whether or not they were receiving chemotherapy, then randomized to either exercise or usual care. While there was no main effect for exercise on sleep in the entire group, the cycling program significantly improved sleep quality in the patients actually receiving chemotherapy. This finding suggests that chemotherapy-induced sleep problems might involve factors uniquely amenable to exercise therapies (Courneya et al., 2012). The inclusion of resistance training has also been shown to improve sleep quality in patients receiving chemotherapy. In a study involving 66 patients with stage IV lung or colorectal cancer, 45% of whom were receiving chemotherapy, Cheville et al. found that participants randomized to an 8-week exercise program involving both strength training and walking reported less fatigue and better sleep quality compared to the usual care group (Cheville et al., 2013).

**Table 2:** Summary of included studies investigating the effects of an exercise intervention on sleep disturbances. Abbreviations: CG, Control Group; IG, Intervention Group.

Study	Participants	Study Design and Follow-up	Exercise Protocol	CIPN measures	Outcomes
Cheville et al. (2013)	Stage IV lung or colorectal cancer patients (44% undergoing chemotherapy) (N = 66) IG: N = 33 CG: N = 33	RCT, outcomes measured at baseline and after 8-week intervention	Type: home-based incremental walking and resistance training (2 sets of 5 exercises, one upper body and one lower body) Frequency: 4 strength and 4 walking sessions/week (total 8 weeks) Intensity: Resistance: use of band that required moderate force but maintained control Aerobic: walking briskly at pace of 1 mile in 20 min Time: strength based on repetitions, walking duration not explicitly stated	Objective: None Subjective: patient-reported scale to assess sleep quality (0–11), Functional Assessment of Cancer Therapy (FACT)-fatigue subscale (0–52)	Following the intervention, IG reported significantly better sleep quality (*p* = 0.05) and less fatigue (*p* = 0.02) than CG. There were significant differences between IG and CG in mean changes in sleep scores (+1.46 vs. –0.10, *p* = 0.002) and fatigue scores (+4.46 vs. –0.79, *p* = 0.03) across the intervention, favoring IG.
Courneya et al. (2014)	Breast cancer patients, ongoing chemotherapy (N = 301) Standard dose of aerobic exercise (STAN): N = 96 Higher dose of aerobic exercise (HIGH): N = 101 Combined dose of aerobic and resistance exercise (COMB): N = 104	RCT, outcomes measured at baseline, 2x during chemotherapy, 1x post-chemotherapy	Type: at-home aerobic or combined aerobic plus resistance training (2 sets of 10–12 reps of nine different exercises) Frequency: 3 days/week (Total duration based on duration of chemotherapy) Intensity: Aerobic: “vigorous” Resistance: 60–75% of estimated 1RM Time: STAN: 25–30 min/session HIGH: 50–60 min/session COMB: 25–30 min of aerobic exercise and 30–35 min of strength training	Objective: none Subjective: Pittsburg Sleep Quality Index (PSQI) (global sleep quality scale 0–21, individual components scale 0–3)	Compared to STAN, HIGH reported significantly better global sleep quality (*p* = 0.039; Cohen’s *d* = 0.22), subjective sleep quality (*p* = 0.028; *d* = 0.26), and sleep latency (*p* = 0.049; *d* = 0.18) following intervention. Compared to STAN, COMB reported borderline significantly better global sleep quality (*p* = 0.085; *d* = 0.19) and sleep duration (*p* = 0.051; *d* = 0.22), significantly better sleep efficiency (*p* = 0.040; *d* = 0.24), and significantly lower proportion of poor sleepers (*p* = 0.045; *d* = 0.20) following intervention. Compared to COMB, HIGH reported significantly better sleep latency (*p* = 0.040; *d* = 0.20) following the intervention.
Courneya et al. (2012)	Lymphoma patients (45% undergoing chemotherapy) (N = 122) IG: N = 60 CG: N = 62	RCT, outcomes measured at baseline and after 12-week exercise intervention	Type: supervised cycle ergometer Frequency: 3 sessions/week (total 12 weeks) Intensity: began at 60% of peak oxygen consumption (VO2_peak_) and was increased by 5% each week to reach 75% by the 4th week Time: began at 15–20 min per session, increased by 5 min per week to reach 40–45 min by the 9th week	Objective: none Subjective: Pittsburg Sleep Quality Index (PSQI)	No significant difference between IG and CG in change in global sleep quality (–0.64, *p* = 0.16; *d* = -0.19) or any component scores. Borderline significant reduction in percentage of poor sleepers favoring IG (–14.2, *p* = 0.086; *d* = -0.27). In planned subgroup analysis, exercise resulted in improved global sleep quality in patients currently receiving chemotherapy (*p* = 0.013; *d* = -0.50), but not in off-treatment patients (*p* = 0.72).
Ligibel et al. (2016)	Metastatic breast cancer patients (42% undergoing chemotherapy) (N = 101) IG: N = 48 CG: N = 53	RCT, outcomes measured at baseline and after 16-week intervention	Type: home based aerobic exercise Frequency: not specified (total 16 weeks) Intensity: “moderate” Time: 150 min/week	Objective: none Subjective: European Organization for Research and Treatment of Cancer Quality of Life Questionnaire Core-30 (EORTQLQ C-30), insomnia subscale	No significant differences between IG and CG in changes in global quality of life (7.0, *p* = 0.17), fatigue (-5.4, *p* = 0.63), or insomnia (-3.7, *p* = 0.22).
Schmidt et al. (2015)	Breast cancer patients, ongoing chemotherapy (N = 81) Endurance group: N = 29 Resistance group: N = 24 CG: N = 28	RCT, outcomes measured at baseline and after 12-week intervention	Type: supervised indoor bike or resistance training (10 strength exercises) Frequency: twice weekly (total 12 weeks) Intensity: Endurance: RPE of 11–14 Resistance: 50% hypothetical 1RM for 20 reps of each exercise Time: 60-minute sessions	Objective: none Subjective: European Organization for Research and Treatment of Cancer Quality of Life Questionnaire Core-30 (EORTQLQ C-30) version 3 BR23 with insomnia subscale, Multidimensional Fatigue Inventory 20 questions (MFI-20)	RT showed significant improvement in QOL (p = 0.011), ET borderline improvement (p = 0.09), and CG no significant change (p = 0.80). There were no significant changes in insomnia or fatigue for either RT or ET groups, as well as the control group.
Tang et al. (2010)	Cancer patients with varying cancer types (14% undergoing chemotherapy) (N = 71) IG: N = 36) CG: N = 35	RCT, outcomes measured at baseline and after 8-week intervention	Type: home-based walking Frequency: 3 days/week (total 8 weeks) Intensity: RPE of 11–13 Time: 30 min each day	Objective: none Subjective: Taiwanese version of the Pittsburg Sleep Quality Index, Medical Outcomes Study Short Form-36 to measure QOL	IG reported significant improvements in sleep quality (p < 0.01) and the mental health dimension of quality of life (p < 0.01) In the IG, improved sleep quality was associated with reduced bodily pain (p = 0.04) and improved mental health over time (p < 0.01)
Wang et al. (2011)	Early stage breast cancer patients, ongoing chemotherapy (N = 72) IG: N = 35 CG: N = 37	RCT, outcomes measured at 24 h prior to surgery (T0), 24 h prior to the first chemotherapy cycle (T1), day of expected nadir (T2), and end of 6-week intervention (T3)	Type: home-based walking (along with strategies to improve self-efficacy) Frequency: 3–5 sessions/week (total 6 weeks) Intensity: HR_max_ of 40–60% Time: 30 min per session	Objective: none Subjective: PSQI, FACT-G version 4 for QOL, FACIT-F fatigue scale	Subjects in exercise group had significantly better quality of life (p < 0.001 & p = 0.011) and less sleep disturbances (p < 0.001 & p = 0.006) over time (p values indicate linear growth rate and quadratic growth rate, respectively) Exercise group had less fatigue than the control group after the intervention period (p = 0.003)
Wenzel et al. (2013)	Prostate, breast, and other solid tumor cancer patients (35% undergoing chemotherapy, others undergoing other cancer treatments) (N = 138) IG: N = 68 CG: N = 58	RCT, outcomes measured at baseline and after each individual’s treatment regimen (ranging from 5 to 35 weeks)	Type: home-based walking Frequency: 5 days/week (total weeks dependent on cancer treatment) Intensity: “brisk” Time: 20–30 min sessions	Objective: none Subjective: PSQI, modified Piper Fatigue Scale (PFS)	Sleep quality for both groups was not statistically different No significant difference in fatigue between groups Regardless of randomization, patients who engaged in more aerobic exercise reported less fatigue (p = 0.035).

While the results of these studies are promising, it is possible that the observed sleep benefits were at least in part attributable to the exercising patients receiving more attention from their care teams, and not necessarily due to the exercise itself. One study sought to evaluate a potential dose response relationship between exercise and sleep outcomes by randomizing 301 breast cancer patients to three different exercise interventions: standard dose of 25–30 min of aerobic exercise 3 days/week (STAN), a higher dose of 50–60 min of aerobic exercise 3 days/week (HIGH), or a combined dose of 50–60 min of aerobic and resistance exercises 3 days/week (COMB). Sleep outcomes were assessed through the Pittsburgh Sleep Quality Index. The HIGH group was found to be statistically superior to the STAN group in the categories of global sleep quality, subjective sleep quality, and sleep latency. The COMB group was also statistically superior to the STAN group in sleep efficiency and overall percentage of poor sleepers, and was borderline superior in the categories of global sleep quality and sleep duration. It is important to note that overall, sleep quality worsened over time in all groups over the course of chemotherapy administration, just to a lesser extent in the groups with higher doses of exercise. Interestingly, this dose-dependent effect, in which HIGH and COMB were found to be more effective than STAN in improving sleep disturbance, was stronger for patients with no comorbidities, those who met aerobic exercise guidelines at baseline, and those who were aerobically fitter at baseline (Courneya et al., 2014). This is consistent with the idea that exercise regimens are most beneficial when they are tailored to an individual’s baseline exercise capacity (i.e. whereas unconditioned individuals may benefit from standard training loads, more highly conditioned individuals have greater capacity to tolerate - and therefore respond to - higher training loads) (Courneya et al., 2014).

In contrast, there have been studies that did not find a significantly beneficial effect of an exercise intervention on sleep quality. These studies done by Ligibel et al. (patients with metastatic breast cancer, 42% receiving chemotherapy) and Wenzel et al. (patients with prostate, breast, or other solid tumors, 35% receiving chemotherapy) did not find any positive effect of exercise with regard to subjective sleep quality (Ligibel et al., 2016) (Wenzel et al., 20132013). Notably, the study by Ligibel et al. investigated sleep quality as one of several dependent variables within comprehensive quality of life questionnaires. Also, its exercise intervention did not produce a significant difference in the amount of exercise performed between the control and intervention groups. The study population of Wenzel et al. included 55.6% prostate cancer patient and 52.4% of patients in the study received radiotherapy alone, compared to 34.9% receiving chemotherapy alone. Thus, most of the patients had not experienced chemotherapy-associated sleep disturbances, limiting the possibility of detecting a difference in sleep quality between the control and exercise group, since deficits were minimal at baseline.

The majority of evidence seems to give credence to the hypothesis that exercise could be a viable therapy to mitigate chemotherapy-associated sleep disturbances, especially if exercise actually increased over baseline. Furthermore, it remains evident that exercise has clear overall health benefits, which may include preventing chemotherapy-induced peripheral neuropathy, and so it should be recommended to cancer patients undergoing chemotherapy. Some suggestions for at-home exercise prescriptions, based on available studies in patients with cancer, are provided in Fig. 1.

## 4 Connection between CIPN and sleep

Patients with neuropathic pain are more likely to develop sleep disorders and the sensation of pain seems to be worsened when patients have poor sleep duration and/or quality (Ferini-Strambi, 2017), leading to the hypothesis that CIPN and sleep may impact each other in a bidirectional relationship. For example, in a large population-based study investigating subjective sleep measures and experimental pain sensitivity, Sivertsen et al. found that frequency and severity of insomnia, sleep onset latency, and sleep efficiency were associated with pain sensitivity in a dose–response relationship (Sivertsen et al., 2015). In a cross-sectional study by Hong et al. of 706 cancer patients, most of whom had received neurotoxic chemotherapy, participants completed questionnaires related to CIPN, psychological distress, and sleep quality. High CIPN scores were associated with poor psychological status and sleep quality even after controlling for several demographic and disease characteristics. CIPN was found to be an independent risk factor for depression, anxiety, and poor sleep quality (Hong et al., 2014). While the cross-sectional nature of these studies allows only for correlations to be examined, it appears that sleep quality and CIPN, especially its pain component, are related. It follows then that if exercise can impact one or both of these variables positively, that it might also indirectly have a positive effect on the other.

## 5 Proposed mechanisms for exercise’s potential benefit in reducing CIPN and improving sleep

Several biological mechanisms have been proposed to explain the relationships between exercise, CIPN, and sleep. In general, exercise counteracts the deleterious effects of chemotherapy on the nervous system by reducing inflammation, promoting brain neuroplasticity, and enhancing pain-inhibitory pathways (Fig. 2). Anti-inflammatory adaptations are also involved in exercise-mediated improvements in sleep disturbances, along with normalization of sleep homeostasis and entrainment of circadian rhythmicity.

On a molecular level, pro-inflammatory signaling and oxidative damage pathways play central roles in CIPN (Brandolini et al., 2019). Chemotherapy-induced increases in intracellular reactive oxygen species (ROS) contribute to nerve degeneration via mitochondrial dysfunction and apoptosis, as well as demyelination due to damage to phospholipids. In response to peripheral nerve damage, Schwann cells, satellite cells in the dorsal root ganglion, and astrocytes and microglia in the spinal cord, release pro-inflammatory mediators such as tumor necrosis factor-Alpha (TNF-A), interleukin-1Beta (IL-1B), IL-6, and IL-8 (Brandolini et al., 2019; Leitzelar and Koltyn, 2021; Costigan et al., 2009). These cytokines lead to neuronal damage both directly through receptor-mediated pathways and indirectly by inciting macrophage recruitment and accumulation (Brandolini et al., 2019). Along with altered neurotrophic signaling by activated microglia, pro-inflammatory cytokines also change neuronal function and increase nociceptive excitability, contributing to hyperalgesia and allodynia (Costigan et al., 2009; Leitzelar and Koltyn, 2021; Scheffer and Latini, 2020).

Exercise appears to alter the inflammatory marker profile and reduce chronic inflammation, and therefore could counteract the development of CIPN. In response to acute bouts of exercise, contracting skeletal muscle produces IL-6, leading to increased levels in circulation. IL-6, when released from skeletal muscle in the context of physical activity, acts to inhibit TNF-A (Starkie et al., 2003) and increase levels of anti-inflammatory cytokines IL-10 and IL-1 receptor antagonist (IL-1RA) (Gleeson et al., 2011) (Scheffer and Latini, 2020) (Fig. 2). While the stress of an acute exercise event leads to transiently elevated levels of pro-inflammatory markers, chronic exercise is associated with adaptive upregulation of anti-inflammatory signaling (Scheffer and Latini, 2020) (Radak et al., 2013). This outcome reduces inflammation-induced pain and nociceptor sensitivity (Scheffer and Latini, 2020), and likely explains why the increase in pro-inflammatory markers seen following nerve injury is blunted by exercise in animal models (Bobinski et al., 2011) (Chen et al., 2012). Chronic exercise also results in upregulation of endogenous antioxidant systems, as well as neuroprotective factors such as brain-derived neurotrophic factor, glial-derived neurotrophic factor, insulin like growth factor, and vascular endothelial growth factor (Radak et al., 2013) (Zimmer et al., 2018) (Park et al., 2015) (Fig. 2).

Another important mechanism in the pathogenesis of CIPN, and in particular the experience of neuropathic pain, involves maladaptive adaptations by the nervous system in response to nerve injury (Costigan et al., 2009). These responses, such as increased ectopic signaling and decreased excitatory thresholds within nociceptive pathways, and inappropriate connections that emerge during nerve regeneration, contribute to the development of neuropathic pain by altering sensory processing (Costigan et al., 2009). Via the process of central sensitization, peripheral nerve injury leads to synaptic changes in the spinal dorsal horn that serve to facilitate signaling within pain pathways (Costigan et al., 2009) (Kleckner et al., 2018). Exercise appears to alter how the brain processes bodily sensations, and it has been suggested that exercise-mediated neuroplastic phenomena in the brain may serve to alleviate neuropathic pain independent of peripheral mechanisms (Kleckner et al., 2018) (Tajerian and Clark, 2017) (Fig. 2).

An additional factor that may be involved in exercise-induced reduction of CIPN includes enhanced functioning of CNS pathways that attenuate nociception. Nerve injury alters monoaminergic and endorphin signaling in the descending pain modulatory system, leading to disinhibited transmission of nociceptive input (Bobinski et al., 2011) (Costigan et al., 2009). It has been suggested that exercise actually may counteract these changes and restore normal inhibitory tone (Fig. 2). Blockade of serotonin (Bobinski et al., 2011) and dopamine (Wakaizumi et al., 2016) signaling in the CNS prevents exercise-induced reductions of hyperalgesia following nerve injury in mice. Within the spinal dorsal horn, inhibitory interneurons producing gamma-aminobutyric acid (GABA) modulate afferent signaling from primary sensory neurons (Costigan et al., 2009). Loss of this inhibitory input has been associated with symptoms of neuropathy, including allodynia and hyperalgesia, and endurance of neuropathic pain (Costigan et al., 2009). Animal models of neuropathic pain demonstrate fewer GABA-producing interneurons in the spinal dorsal horn, suggesting that loss of inhibitory GABA signaling is involved in the pathogenesis of neuropathic pain (Kami et al., 2016). Consistent with this hypothesis, Kami et al. showed that treadmill running after sciatic nerve injury in mice lessened neuropathic pain behaviors and attenuated the loss of GABA-positive neurons in the ipsilateral dorsal horn (Kami et al., 2016).

The biological mechanisms underlying chemotherapy-induced sleep disturbances are less well characterized. Inflammation likely plays an important role, as proinflammatory cytokines associated with cancer and its treatments can cause sleep-wake disturbances, including reduced sleep efficiency and increased sleep latency (Palesh et al., 2012). Additional factors involved in poor sleep quality in cancer patients receiving chemotherapy include hypothalamic–pituitaryadrenal (HPA) axis dysfunctionality and disruptions to circadian rhythms (Palesh et al., 2012). Indeed, sleep disturbances in cancer patients undergoing chemotherapy have been associated with flattened diurnal cortisol cycles (Palesh et al., 2012).

The ability of exercise to improve sleep in this patient population centers primarily around its roles in promoting an anti-inflammatory state and in normalizing sleep homeostatic mechanisms. Exercise-induced reduction in systemic inflammation has been associated with improved sleep quality, particularly in patients with sleep disturbances (Palesh et al., 2012). From a homeostatic standpoint, exercise is a powerful non-photic synchronizer, meaning it can significantly influence the body’s circadian clock (Hower et al., 2018; Palesh et al., 2012). Repeated stimulation of the neuroendocrine system by regular physical activity leads to hormonal changes (e.g. larger reductions in nighttime cortisol levels, increased production of melatonin) that entrain circadian rhythmicity and improve sleep quality (Hower et al., 2018). Another means by which exercise might benefit sleep includes effects on body temperature regulation. According to the thermogenic hypothesis, sleep onset is promoted by a decrease in core body temperature through heat dissipation. Exercise, which increases core body temperatures acutely, can facilitate sleep onset via subsequent promotion of a counterregulatory heat dissipation response (Tang et al., 2010).

In summary, exercise appears to exert its protective effects against CIPN and sleep disturbances via modulation of the inflammatory response, alteration of neural circuitry, and regulation of circadian rhythmicity. While the details of these mechanisms remain incompletely characterized, advancements in our understanding will hopefully allow for more targeted prescription of exercise in the future.

## 6 Conclusions

This review highlights the evidence supporting a potential role of exercise in limiting the morbidity associated with CIPN and sleep disturbance. Chemotherapy plays a pivotal role in ovarian cancer treatment and can lead to problems with quality of life in patients, even if they are cured of their disease, and certainly among the majority who will be receiving chemotherapy for years as part of treatment for a chronic disease process. Clinicians have long known the benefit of exercise but it is difficult to convey that information in a usable manner in terms of advising their patients on a specific exercise program to fulfill recommendations, such as those outlined in Fig. 1.

More randomized controlled trials are needed to assess both the feasibility and efficacy of specific exercise interventions as a legitimate treatment strategy for CIPN, sleep disturbances, and other quality of life issues for cancer patients. One such trial, the Physical Activity and Dietary Intervention in Women with Ovarian Cancer (PADOVA) study, is underway in Amsterdam. PADOVA will investigate the effects of a combined exercise and dietary intervention on women undergoing neoadjuvant chemotherapy for the treatment of ovarian cancer (Stelten et al., 2020). Continued research in this arena will help all clinicians provide the best care for their patients but while awaiting those results, it would be prudent that all oncologists become comfortable and knowledgeable in how to recommend and prescribe specific exercise regimens for their patients.

### CRediT authorship contribution statement

**L. Brett Whalen:** Conceptualization, Data curation, Writing – original draft, Visualization. **W. Zachary Wright:** Data curation, Writing – original draft, Visualization. **Priyanka Kundur:** Data curation. **Siddhartha Angadi:** Writing – review & editing, Supervision. **Susan C. Modesitt:** Conceptualization, Writing – review & editing, Supervision.

## Declaration of Competing Interest

The authors declare that they have no known competing financial interests or personal relationships that could have appeared to influence the work reported in this paper.
