# Supplementary material for: Beneficial effects of exercise on chemotherapy-induced peripheral neuropathy and sleep disturbance: A review of literature and proposed mechanisms
Source: Gynecol Oncol Rep. 2022 Jan 7;39:100927. doi: 10.1016/j.gore.2022.100927 (PMC8761687; doi:10.1016/j.gore.2022.100927)
Supplement: Supplementary data 1 [file mmc1.docx]

Upper and lower body resistance exercises, modeled after Rapid, Easy, Strength Training (REST) Exercises as described by Cheville et al. [20].

Upper body exercises:

- **Biceps curls**: Anchor the band under your feet. Starting with your hands by your sides, bend your arms at the elbows, making sure to keep your elbows close to your body. Finish with your hands close to your shoulders. Hold this position for one full second before returning to starting position.
- **Rows**: Anchor the band around an object at chest height (either standing or sitting). Starting with your arms straight out in front, squeeze your shoulder blades together and pull the band in toward your body. Finish with your hands in close to your sides, near the bottom of your ribcage. Hold this position for one full second before returning to starting position.
- **Pull downs**: Anchor the band above eye level. Keeping your elbows tucked by your sides, start by straightening your arms until your hands are by your sides. Finish the movement by pulling your arms through until your hands are slightly behind your body with your arms kept straight (i.e. no bend in the elbows). Hold this position for one full second before returning to starting position.
- **Bat swings**: Anchor the band at about chest height. Keeping your arms straight and directly out in front of you throughout the movement, rotate your upper body until your arms are pointing in the opposite direction of where the band is anchored (i.e. about 90 degrees of rotation). Hold this position for one full second before returning to starting position.
- **Chest press**: Anchor the band at about chest height. Facing away from the band, start with your hands close to your sides, near nipple level. Push your arms straight out until there is no bend in your elbows, finishing with your hands near eye level. Hold this position for one full second before returning to starting position.

Lower body exercises:

- **Squats**: From a standing position, feet a little wider than shoulder-width apart, start by gradually bending your knees and hips (i.e. in a motion similar to sitting back into a chair), continuing to lower your hips until your thighs are as close to parallel with the ground as you are able. Finish the movement by returning to a standing position.
  - Modification 1: From a standing position, gradually sit back into a chair (or any stable, elevated surface), then stand back up.
  - Modification 2: Standing with your back against a wall, your feet about 12 inches from the wall, start by gradually bending your hips and knees, allowing your back to slide down the wall. Continue to lower until your thighs are as close to parallel with the ground as you are able. Finish the movement by returning to a standing position.
- **Calf raises**: From a standing position, keep your legs straight (i.e. no bend in the knees) and rise onto the balls of your feet, lifting your heels off the ground. Hold this position for one full second before returning your heels to the ground.
  - For added resistance, anchor a band under your feet and grasp the ends.
- **Straight leg steps, front**: From a standing position, keep your leg straight and kick it forward until your hip has bent by about 20 degrees. Hold this position for one full second before returning your leg to the starting position. Perform all repetitions with one leg before switching to the other side.
- **Straight leg steps, sideways**: From a standing position, keep your leg straight and lift it out to the side until your hip has bent by about 20 degrees. Hold this position for one full second before returning your leg to the starting position. Perform all repetitions with one leg before switching to the other side.
- **Straight leg steps, backward**: From a standing position, keep your leg straight and kick it backward until your hip has extended by about 20 degrees. Hold this position for one full second before returning your leg to the starting position. Perform all repetitions with one leg before switching to the other side.
